# Transient Global Amnesia Associated with an Acute Infarction at the Cingulate Gyrus

**DOI:** 10.1155/2014/418180

**Published:** 2014-07-13

**Authors:** Alejandro Gallardo-Tur, Jorge Romero-Godoy, Carlos de la Cruz Cosme, Adriá Arboix

**Affiliations:** ^1^Unidad de Gestión Clínica Intercentros de Neurociencias de Málaga, Instituto de Investigación Biomédica de Málaga (IBIMA), Hospital Universitario Virgen de la Victoria/Universidad de Málaga, Spain; ^2^Servicio de Neurología, Hospital Universitari del Sagrat Cor, Barcelona, Spain

## Abstract

*Background*. Transient global amnesia (TGA) is a syndrome of sudden, unexplained isolated short-term memory loss. In the majority of TGA cases, no causes can be identified and neuroimaging, CSF studies and EEG are usually normal. We present a patient with TGA associated with a small acute infarct at the cingulate gyrus. *Case Report*. The patient, a 62 year-old man, developed two episodes of TGA. He had hypertension and hypercholesterolemia. He was found to have an acute ischemic stroke of small size (15 mm of maximal diameter) at the right cerebral cingulate gyrus diagnosed on brain magnetic resonance imaging. No lesions involving other limbic system structures such as thalamus, fornix, corpus callosum, or hippocampal structures were seen. The remainder of the examination was normal. *Conclusion*. Unilateral ischemic lesions of limbic system structures may result in TGA. We must bear in mind that TGA can be an associated clinical disorder of cingulate gyrus infarct.

## 1. Introduction

Transient global amnesia (TGA) is a self-limited disorder characterized by a sudden, unexplained, short-term memory loss, with preserved consciousness and self-awareness. The patients often exhibit stereotyped behavior, asking the same question over and over again. Total or partial amnesia for the episode persists and there are no other neurological signs and symptoms. In the majority of TGA, no causes can be identified and neuroimaging; CSF studies and EEG are usually normal. The pathophysiology of TGA is unclear and vascular etiology, epileptic, tumorous, or migrainous mechanisms, venous congestion, and psychological disturbances have classically been postulated [1, 2].

Moreover, TGA associated with an acute cerebral infarct has rarely been reported. The patient described here developed TGA resulting from a right small cingulate infarct. To our knowledge, this is the first case that demonstrates that TGA can be associated with acute infarction only in the right cingulate gyrus.

## 2. Case Report

**Figure 1:**
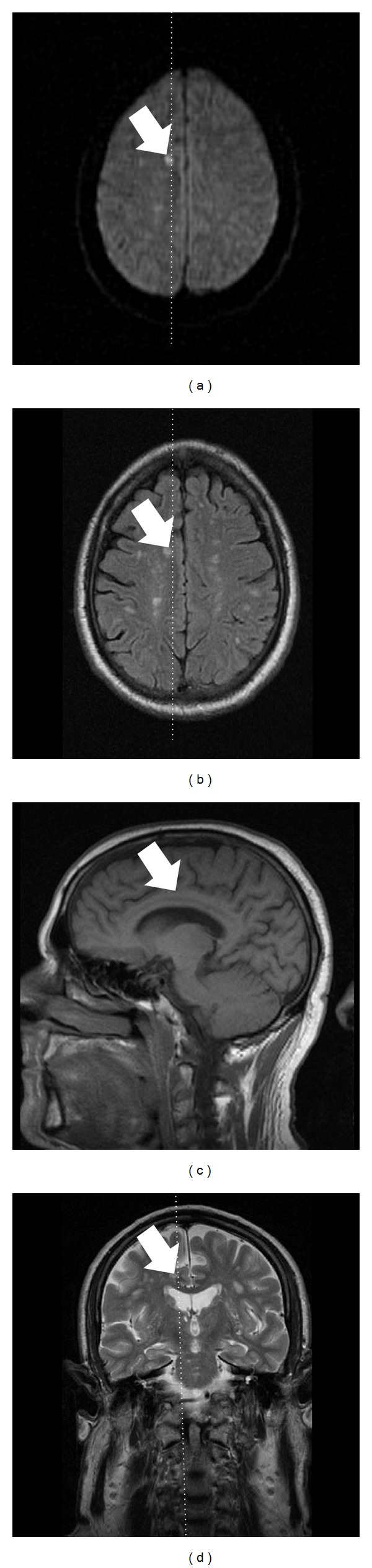
Right small acute cingulate gyrus infarct was found by magnetic resonance imaging on diffusion-weighted images study (a), T2-weighted image ((b) and (d)), and T1-weighted image (c) on 72 hours after onset of transient global amnesia.

A 62-year-old right-handed white male with a history of hypertension and hyperlipidemia was treated at the emergency room. He complained of two similar episodes within the previous 48 hours, consisting in repetitive questions to his wife with face of strangeness: “what time is it?” and other similar questions related to the activities that they had been doing the same day, for every 10 minutes for 2 hours with expressions of confusion. Subsequently, the patient did not remember anything about what happened during the episode. A similar episode happened the following day, and he returned to the emergency room. There was no history of migraine, psychiatric illness, head trauma, epilepsy, or alcohol abuse. There were no other pathological findings in his medical history. He asked the same questions over and over again, showing great difficulties in grasping simple information. All symptoms disappeared after a period of 120 minutes. Immediate and remote memories were preserved but he could not remember what had happened since the beginning of the symptoms and retrograde amnesia was present covering a period of several hours. Other high cerebral functions were preserved. On examination the patient was alert with a blood pressure of 130/80 mm/Hg, regular heart beats of 70 beat/min, and a temperature of 36.8°C. General and neurological examination showed no pathological findings (mRS = 0; NIHSS = 0). A neuropsychological test for memory function performed after the amnesic episode was within normal limits. The Minimental State Examination score was 30/30. The complete hematological screening, routine biochemical profile, urinalysis, thrombophilic, and autoimmunologic blood test, chest roentgenography, 12-lead electrocardiography, Doppler ultrasonography of the supra-aortic trunks, and 24 h Holter monitoring were normal. Brain MRI study on day 3 after TGA showed a 14 mm T2-hyperintense lesion on diffusion-weighted image (DWI) suggesting an acute ischemic infarct on right cingulate gyrus (Figure 1) with mild chronic ischemic periventricular white matter hyperintensities. The hippocampus, thalamus, and basal ganglia appeared normal. Intracranial MR angiography was also normal.

At the 6th and 12th month followup controls the patient remained entirely assymptomatic.

## 3. Discussion

In this case report, we show that a cingulate infarction can cause transient global amnesia (TGA). Cingulate gyrus infarct is unusual and could cause other syndromes like astasia (inability to sit, stand, and walk) and dysarthria, probably as a result of the relationship with vestibular and cerebellar nucleus [3].

As no structural causes can be identified on neuroimaging, the pathophysiology of TGA remains unclear [1, 2]. There is no scientific consensus on the etiology of this syndrome: its origin could be explained as vascular, ischemic, or migrainous. Some authors support the epileptic etiology [1]. However, the most plausible theory to date is that TGA correlates with a transient perturbation of hippocampal function in the cornu ammonis field due to an increased venous pressure, stress, or other nonspecific processes. TGA has been associated with bout of cough [4]. Recent literature shows that increased lactate, calcium, glutamate, and other molecules could cause a metabolic stress on the hippocampal region. These mechanisms could be involved in the perturbation of the memory symptoms due to the vulnerability of the memory-relevant structures of the mesiotemporal region [1, 2]. Recent data from studies that used high resolution MRI (preferentially on a 3T unit) have shown that focal hyperintense lesions correlating to restricted diffusion in the lateral hippocampus can be reliable detected, thus supporting a metabolic hypothesis. There are reported cases of TGA episodes and punctuate lesions in both hippocampi (both bilateral and symmetrical lesions in the same patient) with hyperintense DWI between 4 and 72 hours after onset. Those cases of coetaneous lesions in both hippocampi support the metabolic hypothesis [1]. Epidemiological data suggest that the recurrence rate of TGA is low, but a few patients could have a second episode.

The case reported here of TGA caused by an acute stroke is extremely uncommon. Cerebral ischemia or cerebral hemorrhage has been suspected of being responsible for TGA when causing dysfunction in the limbic system involved in memory (thalamus, hippocampus, amygdale, fornix, mammillary bodies, cingulate gyrus, and frontal cortex).  Table 1 summarizes the stroke typologies gathered in the studies reporting TGA due to acute stroke: thalamus (hemorrhagic and ischemic stroke) [5–7], frontal lobe (hemorrhagic lesion) [8], occipital lobe [9], corpus callosum (ischemic stroke) [10, 11], caudate nucleus (ischemic stroke) [12], cingulate gyrus (hemorrhagic stroke) [13], and hippocampus (ischemic stroke) [14].

We have found another similar case report of TGA involving cingulate gyrus: the study of Yoon et al. [13], which describes the case of a 57-year-old man with TGA associated with acute intracerebral hemorrhage at the cingulate gyrus. Therefore, our case would be the second one reporting TGA related to cingulate acute stroke. However, our patient developed an ischemic cerebral stroke and, to the best of our knowledge, this is the first case which associates TGA with acute infarct of the cingulate gyrus.

Furthermore, it is worth noting that most of the cerebral infarcts which involve exclusively any of the structures of the hippocampo-mammillo-thalamic pathway are lacunar infarcts, mainly from thalamic topography, which may cause a pure sensory stroke or atypical lacunar syndromes [15, 16] while causing the TGA to remain exceptional [5–7, 12, 17].

## 4. Conclusion

TGA is a self-limited anterograde amnesia syndrome that closely correlates with perturbation of the hippocampus. However, there are other structures involved in memory circuits which may also be involved in this entity as the cingulate gyrus. Unilateral ischemic lesions of any limbic system structure may develop TGA. We should bear in mind that TGA can be a presenting symptom of cingulate gyrus infarct.

## Figures and Tables

**Table 1:** Main studies of TGA associated with acute stroke reported in the literature.

First author, year [reference]	Brain topography	Stroke subtype
Bogousslavsky, 1988 [5]	Thalamus, lenticular nucleus	Intracerebral haemorrhage
Gorelick, 1988 [6]	Thalamus	Ischemic stroke
Chen, 1996 [7]	Thalamus	Intracerebral hemorrhage
Jacome, 1988 [8]	Frontal lobe	Intracerebral hemorrhage
Ott, 1993 [9]	Occipital lobe	Ischemic stroke
Saito, 2003 [10]	Corpus callosum	Ischemic stroke
Moussouttas, 2005 [11]	Fornix, corpus callosum	Ischemic stroke
Ravindran Jain, 2004 [12]	Caudate nucleus, hyppocampus, thalamus	Ischemic stroke
Yoon, 2006 [13]	Cingulate gyrus	Intracerebral hemorrhage
Carota, 2012 [14]	Hippocampus	Iscaehmic lesion
Present case, 2014	Cingulate gyrus	Ischemic stroke
